# Too Blind to See the Elephant? Why Neuroscientists Ought to Be Interested in Tinnitus

**DOI:** 10.1007/s10162-021-00815-1

**Published:** 2021-10-22

**Authors:** Marlies Knipper, Birgit Mazurek, Pim van Dijk, Holger Schulze

**Affiliations:** 1 Molecular Physiology of Hearing, Tübingen Hearing Research Centre (THRC), Department of Otolaryngology, Head & Neck Surgery, University of Tübingen, Elfriede-Aulhorn-Straße 5, 72076 Tübingen, Germany; 2 Tinnitus Center Charité, Universitätsmedizin Berlin, Berlin, Germany; 3 Department of Otorhinolaryngology/Head and Neck Surgery, University of Groningen, University Medical Center Groningen, Groningen, The Netherlands; 4 Graduate School of Medical Sciences (Research School of Behavioural and Cognitive Neurosciences), University of Groningen, Groningen, The Netherlands; 5 Experimental Otolaryngology, Friedrich-Alexander Universität Erlangen-Nürnberg, Waldstrasse 1, 91054 Erlangen, Germany

**Keywords:** tinnitus, hyperacusis, parvalbumin positive interneuron, fast auditory processing, stress, attention

## Abstract

A curative therapy for tinnitus currently does not exist. One may actually exist but cannot currently be causally linked to tinnitus due to the lack of consistency of concepts about the neural correlate of tinnitus. Depending on predictions, these concepts would require either a suppression or enhancement of brain activity or an increase in inhibition or disinhibition. Although procedures with a potential to silence tinnitus may exist, the lack of rationale for their curative success hampers an optimization of therapeutic protocols. We discuss here six candidate contributors to tinnitus that have been suggested by a variety of scientific experts in the field and that were addressed in a virtual panel discussion at the ARO round table in February 2021. In this discussion, several potential tinnitus contributors were considered: (i) inhibitory circuits, (ii) attention, (iii) stress, (iv) unidentified sub-entities, (v) maladaptive information transmission, and (vi) minor cochlear deafferentation. Finally, (vii) some potential therapeutic approaches were discussed. The results of this discussion is reflected here in view of potential blind spots that may still remain and that have been ignored in most tinnitus literature. We strongly suggest to consider the high impact of connecting the controversial findings to unravel the whole complexity of the tinnitus phenomenon; an essential prerequisite for establishing suitable therapeutic approaches.

## INTRODUCTION

**Fig. 1:**
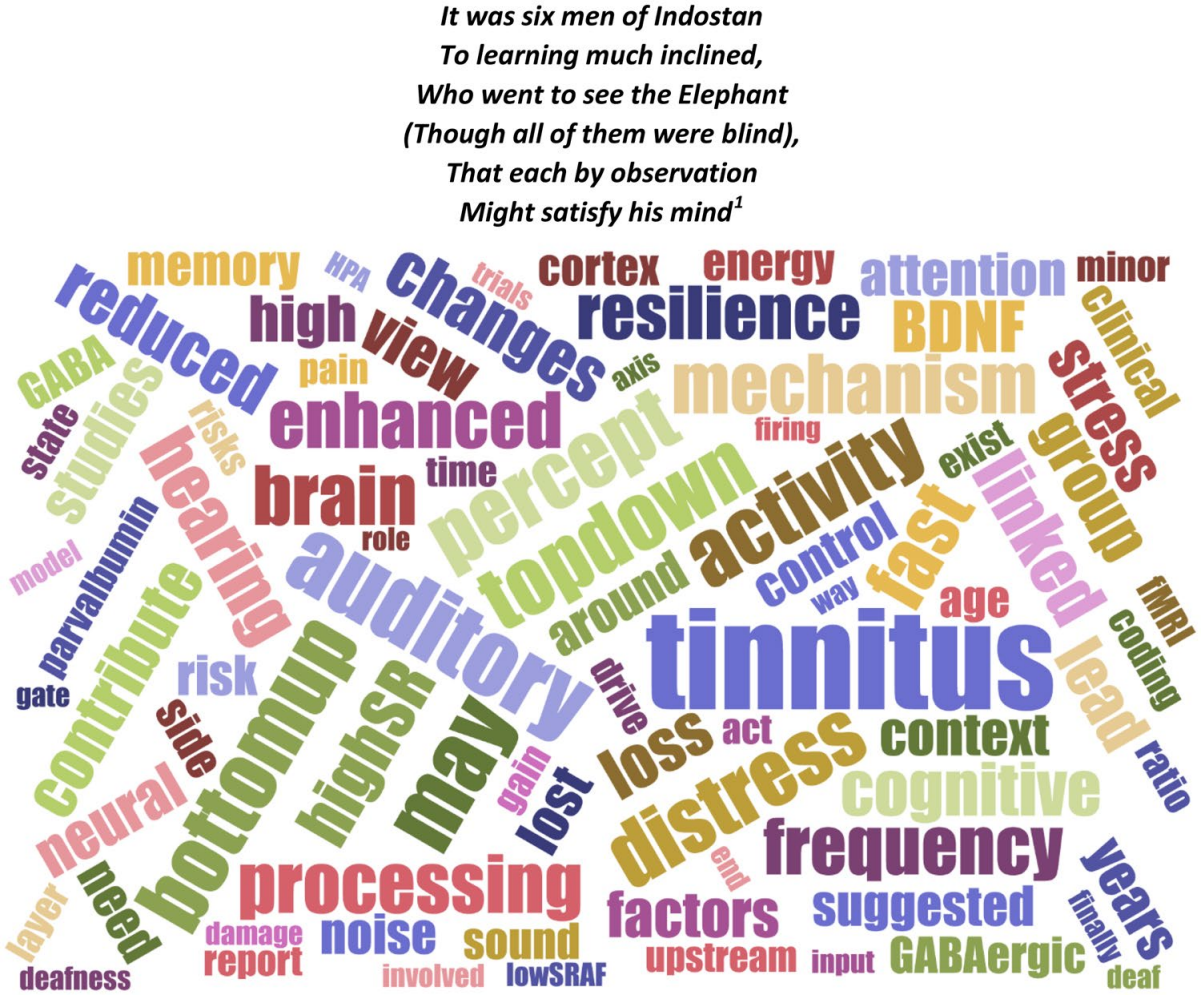
Words dropped during the discussion round at the Annual Mid-Winter Meeting of the Association for Research in Otolaryngology on the 25th of February, 2021 that were automatically selected and ranked by a computer algorithm which analyzed the transcript of the discussion

Questions asked by leading scientists in 2020 around the topic ‘tinnitus’ were the focus of a discussion on the 25th of February, 2021, at the Annual Mid-Winter Meeting of the Association for Research in Otolaryngology which was headed by the authors of the present review. The questions spanned topics around the role of (i) inhibitory circuits, (ii) attention, (iii) stress, (iv) sub-entities, (v) development, (vi) perception, and (vii) successful intervention strategies. During the discussion it was suggested that the ongoing research and modular approach to search for the neural correlates of tinnitus resemble the parable of the ‘blind men and the elephant’, the origins of which have been traced to the Indian subcontinent prior to 500 BCE. We here summarize the questions and answers addressed by the panel, sub-divided into seven categories, and point to the possible existence of a blind spot in the field of tinnitus research. The article should not be understood as an all-encompassing review, but as a reference to the respective research interests of the authors of this manuscript in certain areas. Although at the end of this article a single approach is outlined as an apparent "main concept" worthwhile to focus on in an interdisciplinary, international effort, we emphasize that this might possibly hold only for a minority of tinnitus sufferers. The likely existing great variety of tinnitus forms, however, require a great deal of effort to find markers that allow us to better distinguish between the different forms of tinnitus. Within this context we here pinpoint new questions and future tasks for improved tinnitus therapies, and hope to inspire a cohesive motivation to consider viewpoints that were previously less regarded, but when different disciplines collaborate may allow to uncover the neural correlate of tinnitus as a decipherable phenomenon that can ultimately be therapeutically addressed (Fig. 1).

It was six men of Indostan

To learning much inclined,

Who went to see the Elephant

(Though all of them were blind),

That each by observation

Might satisfy his mind^1^

### Topic 1: Contribution of Bottom-up and Top-down Inhibitory Circuits to Tinnitus?

The initial discussions centered around the possibility that hearing loss or peripheral deafferentation, as a *bottom-up* contributor, may be linked to a *top-down* mechanism which leads to a tinnitus percept. How can a minor change in bottom-up activity due to deafferentation trigger neural gain or the bursting of epileptic firing that, through a possible qualitative shift in GABAergic activity, may in the end lead to a tinnitus percept? Can bottom-up and top-down activity changes induce tinnitus percepts independently, or does bottom-up neural activity trigger a top-down mechanism? There was agreement that a bottom-up mechanism, independent of whether it is caused by hearing loss, cochlear damage, or deafferentation, will be linked to a top-down modulation through the limbic system and attentional circuits. It was suggested that this ‘two-step’ process, likely triggered through cochlear damage and reduced auditory input, alters upstream neurotransmitters such as, GABA and changes signal processing via, for example, stochastic resonance (Krauss et al. 2016); this might change the upstream coding underlying normal perception. On that basis, it is unimportant whether the bottom-up mechanism acts through increased spontaneous activity, increased synchrony, increased bursting, adaptive stochastic resonance or neural gain: the crucial open question would be to understand how the ‘higher-order’ top-down mechanism induces a chronic manifestation of tinnitus over time.

A long-standing discussion of numerous tinnitus models deals with the idea of a two-step bottom-up and top-down mechanism during tinnitus generation, albeit from different perspectives: (i) a bottom-up deprivation that leads to a top-down modulation failure of auditory gating and noise cancellation, or an impaired central gate keeper that leads to a tinnitus percept (Leaver et al. 2011; Rauschecker 2010; Rauschecker et al. 2014), (ii) a bottom-up tinnitus development linked to hearing loss that can lead to a tinnitus percept independently of any top-down mechanism. Here, top-down tinnitus results from a network problem between the auditory and non-auditory brain areas, including the pregenual anterior cingulate cortex (Vanneste et al. 2018a, b) linked to thalamocortical dysrhythmia (Vanneste et al. 2018a, b, 2019), (iii) a bottom-up tinnitus precursor that is normally ignored as imprecise evidence against the prevailing percept of ‘silence’ (Sedley et al. 2016) and that is amplified through a top-down mechanism through focused attention (Hullfish et al. 2019; Sedley et al. 2016), fear, anxiety, or stress (Jastreboff et al. 1996), or through a combination of these facilitators. Influences on the individual tinnitus severity depend on the context of their culture and experience (Searchfield 2014).

Thus, if we ask under which circumstances minor cochlear deafferentation − as a starting point in bottom-up tinnitus development − might change GABAergic strength in affected circuits to such a level that it could profoundly alter top-down circuits such that upstream coding of normal percepts is permanently altered, we may already have the answer: fundamental differences in central processing and sound coding are expected between the approx. 40 % of auditory fibers with lower spontaneous discharge rate and high thresholds (low-SR-AF) and the 60 % of auditory fibers with high spontaneous firing rates and lowest thresholds (high-SR-AF) (Bharadwaj et al. 2015; Liberman 1978). Up to now, the low-SR-AF are assumed to be particularly vulnerable to acoustic overexposure and ageing (Liberman and Kujawa 2017; Wu et al. 2019). This auditory fiber type has been suggested to drive shifts in inhibitory responses through enhanced central neural gain that are possibly involved in tinnitus (Schaette and McAlpine 2011; Shore et al. 2016). Alternatively, a critical diminution of specifically fast (high-SR) auditory fiber processing has been suggested to be causally linked to tinnitus through shifts in tonic inhibitory responses that lead to a loss of central neural gain (Hofmeier et al. 2018, 2021; Knipper et al. 2020; Möhrle et al. 2019; Refat et al. 2021; Rüttiger et al. 2013; Singer et al. 2013).

As such, the community might reconsider the lost function of high-SR, low threshold neurons in defined tinnitus frequency channels as a bottom-up mechanism and as a rationale to explain numerous observations:

(i) Reduced auditory brainstem and cortical responses in tinnitus (Bramhall et al. 2019; Hofmeier et al. 2018; Koops et al. 2020), reduced functional connectivity in the auditory pathway (Boyen et al. 2014; Lanting et al. 2014), and increased latencies in tinnitus (Hofmeier et al. 2018, 2021; Majhi et al. 2019; Milloy et al. 2017; Möhrle et al. 2019) in connection with decreased myelination of the auditory pathway in tinnitus patients (Koops et al. 2021). The latter was observed by fixel^2^-based analysis in which a tinnitus-related atrophy of the left acoustic radiation near the medial geniculate body was the first evidence of a decrease in myelination of the auditory pathway (Koops et al. 2021). This would suggest a more profound peripheral deafferentation of larger-diameter, high-SR auditory fibers that are more robustly myelinated (Bauer et al. 2007).
(ii) Increased epileptic bursting (Jastreboff et al. 1996), adaptive stochastic resonance (Krauss et al. 2016, 2017, 2018b), and excessive neuronal synchrony in the auditory cortex in tinnitus (Eggermont and Tass 2015; Noreña and Farley 2013) may all be explained through a loss of tonic inhibition that would result in a rapid increase in bursting or epileptic firing and reduced signal-to-noise ratios (Duguid et al. 2012; Hsieh et al. 2017; Rossignol et al. 2013).
(iii) Diminished activity of the tonic fast-spiking parvalbumin (PV)+ interneuron networks would, in turn, be expected to be linked to enhanced baseline spontaneous gamma power (Mamashli et al. 2017), and thus explain enhanced spontaneous gamma oscillations in tinnitus patients (Ortmann et al. 2011; Vanneste et al. 2018b, 2019; Weisz et al. 2007). This would also be compatible with enhanced variance in stimulus-induced responses and lower signal-to-noise ratios that contribute to tinnitus (Zeng 2020) thereby reducing the gate keeping or noise cancellation linked to dysrhythmia (Leaver et al. 2011; Rauschecker 2010; Rauschecker et al. 2014; Vanneste et al. 2019), reducing functional connectivity in the auditory pathway (Boyen et al. 2014; Lanting et al. 2014) (for a review see (Knipper et al. 2020)).
(iv) Increased susceptibility to a clinical manifestation of tinnitus linked to enhanced tinnitus-related distress, as observed in patients with brain-derived neurotrophic factor (BDNF) Val^66^ Met polymorphism (Vanneste et al. 2021). This single-nucleotide polymorphism (SNP) in the BDNF gene (substitution of valine to methionine) leads to a decrease of activity-dependent intracellular trafficking and secretion of the neuronal BDNF (Chen et al. 2004) which is required for balanced hypothalamus-pituitary-adrenal (HPA) axis control (Jeanneteau et al. 2019). During enhanced memory-linked auditory adjustment processes, fast (high-SR) auditory processing is predicted to drive activity-dependent BDNF translation and secretion in the hippocampus (Eckert et al. 2021; Marchetta et al. 2020; Matt et al. 2018).
(v) The lower risk of tinnitus in congenital deafness (Eggermont and Kral 2016) and in congenital unilateral deafness (Lee et al. 2017), and the elevated risk of tinnitus in the implanted ear of bilaterally or unilaterally deaf children when CI are switched off (Baguley and Atlas 2007; Chadha et al. 2009; Ramakers et al. 2015) (for a review, see (Knipper et al. 2020)).
(vi) Finally, the significantly higher gray matter volume in the lingual gyri observed in hearing loss in the tinnitus group compared to the hearing loss group without tinnitus (Koops et al. 2021), can also be taken as an indicator of the loss of fast auditory processing in tinnitus. The lingual gyrus, also known as the medial occipito-temporal gyrus, is linked preferentially to processing vision (Kozlovskiy et al. 2014). This observation is particularly exciting, as it may point to a loss of clustering of auditory and visual modalities in tinnitus, and provide a rationale for the exuberant connections between the visual and auditory cortex found, for example, in deaf cats, together with an increased visual responsiveness in the auditory cortex (Land et al. 2016). It may support the view that within the frequency channel of the tinnitus percept, the mature, fine-grained wiring that is based on fast (tonic) inhibitory circuits which develops with auditory experience is reversed towards an immature pattern (Knipper et al. 2020). This concept should lead to reconsideration of reported tinnitus-related increases in the connectivity of the left lingual gyrus with the left auditory cortex (Hinkley et al. 2015) and decreased connectivity of the left lingual gyrus with the auditory resting-state network (Schmidt et al. 2013, 2017).

#### Future Tasks to See and Test for a Possible Blind Spot

The contribution of fast (high-SR) auditory fiber dysfunction as the trigger of a bottom-up mechanism that, through altered tonic inhibitory strength, leads to top-down activity changes that promote the development of a tinnitus percept could be tested in future studies. A possible approach would be the use of pharmaceutical drugs that either antagonize tonic or phasic GABA receptor units in behavioral animal models of tinnitus, followed by clinical trials. Additionally, morphometric measurements of auditory nerve density could enable to examine the contributions of large-diameter high-SR auditory nerve fibers in tinnitus patients. Moreover, such contributions could then be analysed for associations with changes in top-down cortical oscillatory signatures and dysrhythmia (Lee et al. 2020; van Gendt et al. 2012) or distress and functional-network topology changes, as described in the context of tinnitus (Yoo et al. 2021).

### Topic 2: Contribution of Attention to Tinnitus and Its Relation to Long-term Habituation and Resilience

There are numerous studies that report impaired or altered *executive attention*, *selective attention*, and working memory in chronic tinnitus (Khan and Husain 2020; Mazurek et al. 2019; Mohamad et al. 2016; Nagaraj et al. 2020). Moreover, no doubt exists that the distress accompanying tinnitus influences general and crystallized intelligence and executive function (Mohamad et al. 2016; Neff et al. 2021). Tinnitus appears to be a symptom of a hyperactive cognitive control network: In this maladaptive network, the negative emotion experienced during tinnitus leads to a consumption of cognitive resources that are typically required for proper functioning of auditory working memory. This imbalanced cognitive control contributes to increased vigilance to the tinnitus tone (Mazurek et al. 2019; Trevis et al. 2016). This concept was confirmed, by previous studies of (Brozoski et al. 2019), who observed that in rats, behaviorally-evidenced tinnitus promotes an increased vigilance to the tinnitus percept, while attention to an auditory-specific task is diminished. In this connection, *long-term habituation* during tinnitus might be associated with a gradual decline of such ‘negative emotions’, or with a decline of factors that contribute to these negative emotions (Elarbed et al. 2021; Luan et al. 2019; Mazurek et al. 2019; Nagaraj et al. 2020).

The contribution of cognitive and perceptual load should also be discussed in this context (Khan and Husain 2020). Perceptual load-tinnitus is an undesired stimulation of the auditory processing pathway, and constant perception of tinnitus affects the cognitive load. Within the same framework, the possible causes of *tinnitus resilience* could be the same as those that contribute to habituation to tinnitus. In general, resilience is the ability to cope with critical situations through the use of personal and socially mediated resources. Thus, factors for resilience are positive emotions, socio-environmental factors, cognitive flexibility, active coping style, and exercise (Faye et al. 2018). Negative or positive environmental or hereditary conditions, differences in early life-stress, or chronic illness leading to depression or anxiety, and differences in temperament or personality, contribute to the so-called ‘*emotional health*’ that influences tinnitus and span degrees from complete tinnitus resilience to a high risk for tinnitus (Aydin and Searchfield 2019; Marks et al. 2019; Searchfield 2014; Searchfield et al. 2020). On the clinical side, *high resilience* in tinnitus patients is associated with less depression, less anxiety and fewer somatic symptoms, and the relationship between resilience and tinnitus distress is mediated by emotional health (Wallhäusser-Franke et al. 2014). In this sense, the lack of tinnitus *habituation* could be explained as a result of a reduced resilience strategy. Here, for example, the absence of hereditary contributors to enhanced distress, which have been shown to increase susceptibility to tinnitus (Amanat et al. 2020; Lopez-Escamez and Amanat 2020; Ruan et al. 2018; Szczepek et al. 2019), including the BDNF Val 66 Met polymorphism (Vanneste et al. 2021), have to be regarded in the context of a possible contribution to tinnitus resilience and long-term habituation.

#### Future Tasks to See and Test for a Possible Blind Spot

The community of tinnitus researchers may reconsider how individual environmental or hereditary differences in attentional/cognitive brain states could contribute to suffering more or less from enhanced ‘noise’ in tinnitus frequency channels that have lost contrast-amplification after reduced fast (high-SR) auditory processing. The strong relation of fast (high-SR) auditory processing as a bottom-up mechanism with attentional/cognitive brain states (Review (Knipper et al. 2020)) needs urgent examination. As additional factors that might directly contribute to hereditary or environmental differences in individuals’ and thereby contribute to either enhanced or decreased habituation or resilience to tinnitus, we suggest to consider also those factors that promote the metabolic fatigue of fast auditory processing. This would include conditions that diminish the function of fast *PV* + *interneurons (PV* + *IN)* activity (Kann et al. 2016), which require a high action-potential (AP)-related energy budget to maintain high-frequency activity and fast temporally precise transmission (Hu et al. 2018).

### Topic 3: Contribution of Stress to Tinnitus and Its Link to the Limbic System and Environmental Factors

The list of studies that demonstrate a close relationship of distress and tinnitus is increasing (Boecking et al. 2021; Durai et al. 2018; Elarbed et al. 2021; Park et al. 2020; van Munster et al. 2020). Numerous studies document altered evoked or resting state BOLD fMRI or EEG activity in tinnitus patients, particularly in those brain regions or networks that are involved in attention, distress, and memory functions (Kandeepan et al. 2019; Mohsen et al. 2019; Pattyn et al. 2016; Vanneste et al. 2021; Yoo et al. 2021). The strong contribution of distress culminates in evidence that insomnia, hearing distress, and anxiety are the best predictors of tinnitus severity, and are indeed stronger predictors than any demographic factors (Beukes et al. 2021; Crönlein et al. 2016).

But what do we already know about the mechanism by which distress influences tinnitus? It is important to distinguish the contribution of oxidative stress, stressful acoustic trauma, or mental stress, which has not yet been considered adequately. All forms of stressful events may eventually accumulate to an imbalance within the HPA axis, leading to differential activation of glucocorticoid and mineralocorticoid receptors, and thereby contribute to chronic tinnitus (Kraus and Canlon 2012; Mazurek et al. 2012; Simoens and Hébert 2012). Enhanced tinnitus-related distress, as observed in patient groups with BDNF Val^66^ Met polymorphism (Vanneste et al. 2018a) would be compatible with BDNF bridging glucocorticoid effects on brain networks through BDNF driven phosphorylation of glucocorticoid receptor (Jeanneteau et al. 2019). Impaired *glucocorticoid receptor* (*GR*) phosphorylation following a reduced activity-dependent BDNF recruitment has been shown to lead to impaired long-term memory retention and deficits in forming postsynaptic dendritic spines after, for example, motor-skill training (Arango-Lievano et al. 2019). Thus it is conceivable that, under healthy conditions, fast (high-SR) auditory fiber processing may recruit activity-dependent BDNF to energize contrast amplification and distress levels (Knipper et al. 2020; Matt et al. 2018).

Currently we do not have a good explanation for the potential differential – or combined − impact that acoustic trauma and stress have on different tinnitus groups, which can be clearly distinguished by their distress response. The analysis of 1228 patients led to four distinct patient phenotypes: divided into (i) an ‘avoidant group’, with few affective or psychosomatic symptoms and less tinnitus distress, (ii) a ‘psychosomatic group´ with profound psychosomatic, emotional, and somatic burden, risk of depression and anxiety, and reduced quality of life, (iii) a ‘somatic group’ with physical symptoms that create distress or underlying medical conditions and higher somatic complaints, like pain or headache, and (iv) a ‘distress group’ with a high level of passive stress and physical exhaustion, anxious depressed mood, these patients are often younger and with correlations of neuroticism and anxiety (Bartels et al. 2010; Niemann et al. 2020).

#### Future Tasks to See and Test for a Possible Blind Spot

We need to understand how bottom-up mechanisms may be linked to the role that distress plays in tinnitus − which has been judged to be the ‘best predictor of tinnitus severity’ (Beukes et al. 2021; Crönlein et al. 2016). Here, BDNF signaling needs to be considered as a potential bridge linking bottom-up changes in tinnitus with an altered distress network. Deficits in BDNF driven GR phosphorylation and LTP retention should be considered in the context of enhanced tinnitus-related distress in patients suffering from the BDNF Val66Met polymorphism (Vanneste et al. 2021).

### Topic 4: Contribution of Non-identified Sub-entities of Tinnitus to Current Controversial Views on the Neural Correlate of Tinnitus

Previous studies analyzing patients with tinnitus and hyperacusis (T+H group) and patients without hyperacusis (T-group) revealed evidence for the existence of tinnitus subcategories (Hofmeier et al. 2018, 2021). When tinnitus co-occurred with hyperacusis, stimuli-evoked increases in sound-evoked, supra-threshold ABR amplitudes and fMRI BOLD responses in the medial geniculate body (MGB) as well as in the auditory cortex were found (Hofmeier et al. 2021; Koops and van Dijk 2021; Refat et al. 2021). While a higher sound-evoked fMRI BOLD activity in cortical and subcortical auditory structures was observed in the tinnitus group with hyperacusis compared to the tinnitus group without hyperacusis, an enhanced response to sound was not found in the tinnitus frequency regions (Koops and van Dijk 2021). On the other hand, in tinnitus patients without hyperacusis the auditory responsiveness, including spontaneous and evoked fMRI BOLD responses was reduced (Hofmeier et al. 2021; Refat et al. 2021). Not only the annoyance, tinnitus loudness and bilateral tinnitus are higher in the T+H group (Hofmeier et al. 2021; Ralli et al. 2017; Refat et al. 2021; Schecklmann et al. 2014), but the co-occurrence of tinnitus with hyperacusis also leads to an increase of tinnitus duration over time (Refat et al. 2021; Vielsmeier et al. 2020). Moreover, when chirp stimuli of different frequency spectra were employed (Hofmeier et al. 2021), the results pointed to reduced auditory response activity in T-groups to higher-frequency stimuli and to enhanced auditory response patterns to lower-frequency stimuli in T+H groups (Hofmeier et al. 2021). The more widespread signal amplification process in patients with tinnitus and hyperacusis is supposed to proceed through an excessive thalamo-cortical activity that may trigger an excitation spread to limbic and pain regions, and ultimately results in an over-attention to increased loudness at all sound frequencies (Hofmeier et al. 2021; Koops et al. 2020). It is conceivable that the enhanced distress levels in T+H patients (Hofmeier et al. 2021; Ralli et al. 2017; Refat et al. 2021; Schecklmann et al. 2014) affect wider frequency ranges, including the pain network (Hofmeier et al. 2021; Refat et al. 2021) and contribute directly to the enhanced annoyance to the ‘noise’ in higher-frequency tinnitus channels.

Overall, the hypothesis that sub-entities of tinnitus exist, and can possibly co-occur in different frequency channels, needs to be validated in larger cohort groups.

We may speculate that even controversial findings of deficits of speech-in-noise intelligibility of tinnitus patients may have their origin in different prevalence of sub-entities of tinnitus. Accordingly, a study of (Zeng 2020) did not find any differences in speech-in-noise intelligibility using a group of young adults (mean age 22.6 years) as a normal-hearing control compared to a heterogeneous group of 45 adults (mean age 44 years) with chronic tinnitus (Zeng 2020). In contrast, (Bureš et al. 2019), who found a poorer ability to detect tones in noise and a higher sensitivity to intensity changes and interaural time differences, used a more harmonized group of 51 tinnitus subjects aged around 66 years, and 68 controls around 69 years. It is feasible that a difference in the prevalence of patients with T+H co-occurrence between both studies explain the difference in speech-in-noise intelligibility between the groups.

#### Future Tasks to See and Test a Possible Blind Spot

The current evidence suggests that hyperacusis and tinnitus pathologies may co-exist in parallel frequency channels of the bottom-up auditory pathways. There is an urgent need to consider that in patients with tinnitus and hyperacusis parallel bottom-up changes exist that differentially affect top-down circuits (Eggermont 2021; Martel and Shore 2020) this hypothesis needs to be validated in larger cohort groups and through multi-center clinical trials.

### Topic 5: Contribution of Maladaptive Information Transmission to Tinnitus?

This question may need to be considered in the context of the brain’s overall ability to constantly try to optimize information transmission from the periphery into the brain (Krauss et al. 2016, 2017, 2018a). In the various disciplines in neuroscience, the optimization of information transmission from the periphery into the brain can be (i) realized through *stochastic resonance*, where added neuronal noise lifts *spontaneous firing rate (SFR)* above the threshold, causing a sensory percept (Krauss et al. 2016; Schilling et al. 2021; White et al. 2019). (ii) Alternatively, based on the *Bayesian model*, the brain is conceived as a prediction machine that informs its memory-based predictions through sensory updating (Hemmer et al. 2015). In this view, tinnitus is the result of a prediction error between the predicted and the actual auditory input. The decrease in sensory updating is reflected by decreased alpha activity, while the prediction error is believed to result in altered theta-gamma and beta-gamma coupling (De Ridder et al. 2015; Durai et al. 2019; Hullfish et al. 2019; Mohebbi et al. 2019). Both models may be covered in another (iii) view that the information transfer from the periphery into the brain operates in a so-called ‘reverberating regime,’ (Cramer et al. 2020; Wilting et al. 2018). This describes an information process that, for example, enables cortical networks to interpolate between the asynchronous-irregular and the critical state by small changes in effective synaptic strength or by the excitation-inhibition ratio (Cramer et al. 2020; Wilting et al. 2018).

In these information processing models, we may miss determinants that define (i) the thresholds for action potentials lowered by stochastic resonance, (ii) the set point or threshold to which predictions are typically made (memory or detection threshold?), (iii) the baseline at which the system reverberates.

#### Future Tasks to See and test a Potential Blind Spot

Considering tinnitus as a diminution of fast (high-SR fiber dependent) auditory processing (Knipper et al. 2020; Zeng 2020), the baseline signal-to-noise ratio would be lost in affected frequency channels. In this view, the brain’s advantage having tinnitus in deprived frequency channels would be to lower the energy budget of the brain. The reversal from a mature fast-processing circuit to a presumably immature state within the affected frequency channels would prevent further metabolic fatigue. Thus, fast inhibitory PV+ interneurons and fast (high-SR) auditory processing (Knipper et al. 2020), comprise of only 2.6–4.6 % of interneurons - but require 14–25 % of the total AP related energy budget of the brain (Hu et al. 2014, 2018). This might explain the high vulnerability for metabolic fatigue of PV+ interneurons (Kann et al. 2016). The evolutionary gain in separately clustering sensory modalities to enhance fast information processing and sensory-specific acuity may create a risk of losing it. On a fast time scale, a mechanism like stochastic resonance, which constantly optimizes information transmission from the periphery to the brain, would improve auditory processing at the cost of generating a tinnitus percept. In that view, tinnitus would be a side effect of the brain’s effort to improve hearing (Gollnast et al. 2017).

### Topic 6: Contribution of Deafferentation to a Tinnitus Percept?

It is still unclear how, through altered GABAergic circuit strength or differential tonic or phasic GABA receptor activation, a bottom-up activity might lead to a tinnitus percept? Bottom-up activity must change activity spreading from the thalamus through a canonical propagation to the different cortical layers, from layer IV to supra-granular layer I–II, and subsequently through a top-down mechanism from the infra-granular layer V/VI to the limbic system (Norena et al. 2021), finally leading to a conscious sound percept. Here, the Bayesian models, including predictive coding, attentional modulation and cortical oscillatory band activity, as neurophysiological substrates for auditory predictions, were suggested to contribute through incomplete top-down processing during auditory scene analysis to the conscious tinnitus percept (Durai et al. 2018; Hullfish et al. 2018). It has been speculated that during the perception of tinnitus, spatio-temporal activity patterns in the auditory cortex must be specific to the quality of the percept, and different from patterns that may be recorded during silence but without any phantom percept (Krauss et al. 2018b).

The discussion included the idea that in a chronically perceived tinnitus percept, minor undetectable changes may also contribute, and propagate, possibly as an adaptive response to a transient signal that has occurred only for a few milliseconds within the bottom-up path. In an attempt to illustrate the scenario of a host of existing tinnitus researchers currently hypothesizing several hundreds of different tinnitus decisive factors, the example of "*the Blind men and the Elephant*" was brought up:

Blind to see the coherent whole behind a tinnitus percept, being too fixated on one individual part.

#### Future Tasks to See and test for a Possible Blind Spot

Here, we dare to suggest one factor that the tinnitus community may still be too ‘*blind*’ to, and that could explain most of the different existing tinnitus models and theories, to wit: the observation that the conscious percept of an auditory stimulus requires a proper maturation of a baseline. On top of this baseline, the auditory stimuli can be facilitated via integration into a fronto-striatal contrast amplification circuit to lead to a percept (Irvine 2018; Oxenham 2018). When we assume that the baseline is lost in ascending bottom-up circuits in distinct frequency channels (Knipper et al. 2020), the top-down circuit will now amplify the ‘enhanced noise’ that is generated as a result of lost contrast amplification in the affected region. The intensity of the ‘amplification process’ may, in turn, heavily depend on the individual’s ‘emotional stage’.

### Topic 7: Future Therapy Approaches on the Basis of Ongoing Controversies About the Neural Correlate of Tinnitus

The strong contribution of distress as the best predictor of tinnitus severity (Beukes et al. 2021; Crönlein et al. 2016) may explain why cognitive behavioral therapy (CBT) has the highest effectiveness in the therapy of chronic tinnitus (European Guideline) (Aazh et al. 2019; Cima et al. 2019). The numerous studies that report stress as a co-factor for tinnitus (Brüggemann et al. 2017; Knopke et al. 2017; Ramakers et al. 2015) justify cognitive behavioral therapy as the currently most effective therapy with the best reduction of tinnitus burden.

Besides the gold standard of rather unspecific CBT, which has been propagated for many years, the concept of bimodal neuromodulation has currently gained attention in tinnitus research through various studies that apparently provide surprisingly good results (Conlon et al. 2020; Riffle et al. 2020). Regarding the contributing factor of general health on tinnitus, efforts to reduce tinnitus by diet (Spankovich and Le Prell 2019) or physical activity (Carpenter-Thompson et al. 2015; Michiels et al. 2016) need further thought. The benefit of invasive tinnitus treatment may outweigh its risks, but with one exception: the extraordinarily invasive method of cochlear implantation. Indeed, increasing evidence suggests that tinnitus can be silenced in most of the implanted tinnitus patients with deafness or severe hearing loss (Baguley and Atlas 2007; Kleine Punte et al. 2013; Knopke et al. 2017; Li et al. 2019; Mertens et al. 2018; Pillsbury et al. 2018; Tyler et al. 2008). This would, of course, still not justify cochlear implantation in tinnitus patients that have little or no hearing loss. A brainstem auditory implant would possibly be beneficial in such cases (Van Den Berge et al. 2019).

The potential of vagus nerve stimulation (VNS) to drive neural plasticity to reduce or eliminate the neural drivers of ongoing tinnitus, although recently judged as a successful approach (De Ridder et al. 2020), may have too many side effects. Effects of pairing of the vagus stimulation with non-tinnitus or tinnitus-matched sounds still has to be determined (De Ridder et al. 2020).

Despite their potential to silence intermittent tinnitus under distinct conditions, other therapies, including, for example, lidocaine, (Vielsmeier et al. 2021), may have too severe side effects. These include psychotropic effects, or effects on blood pressure (Tran and Koo 2014), and their benefits do not seem to outweigh their risks.

#### Future Tasks to See and Test for a Possible Blind Spot

As noted previously, it may be conceivable to work on strategies that specifically trigger stochastic resonance. Alternatively, acoustic stimulation may be re-considered, consistent with previous findings that reported the suppression or relief of the tinnitus percept by electric-acoustic stimulation or by hearing aids (Kleine Punte et al. 2013; Knopke et al. 2017; Li et al. 2019; Mertens et al. 2018; Peters et al. 2020; Pillsbury et al. 2018; Searchfield et al. 2010; Shekhawat et al. 2013).

## CONCLUSION

We suggest that the tinnitus percept resulting from the amplification of ‘enhanced noise’ that is generated as a result of lost contrast amplification subsequent to a loss of fast (high-SR) auditory processing is compatible with numerous apparently controversial findings. In this view certain forms of tinnitus may result from loss of fast (high-SR) auditory fibers and subsequent diminished capacity to suppress intrinsic noise levels in affected frequency channels through PV-IN driven fronto-striatal contrast amplification circuits. This signal is required to energize gate keeping, contrast amplification, neural gain, and adjustments, but when reduced as a result of diminished fast (high-SR) auditory fiber processing, would entail dysfunctions in affected frequency channels that themselves result in elevated noise levels and tinnitus percept. Tinnitus percept is amplified through increased vigilance to the noise following imbalanced cognitive control and lost contrast amplification. Tinnitus percept is amplified through increased vigilance to the noise following imbalanced cognitive control and lost contrast amplification. The tinnitus loudness and burden may differ in individuals, depending on their individual ‘emotional stage’ and distress level. Important to emphasize that this viewpoint may hold only for a small minority of tinnitus sufferers, regarding that likely a great variety of tinnitus forms exists. The authors are however convinced that the search for the most comprehensive possible concrete therapy forms is doomed to failure at least as long as we do not succeed in enabling a better clinical characterization of single forms of tinnitus in patients. In a unified effort across the tinnitus community, tinnitus research could focus on examination of this suggested concept, and find new curative approaches to silence tinnitus.

## Footnotes

1 *Saxe, John Godfrey. "The Blind Men and the Elephant". The poems of John Godfrey Saxe. p. 260.*

2 Fixel refers to a specific fibre bundle within a specific voxel.
